# Pollen Elicits Proboscis Extension but Does not Reinforce PER Learning in Honeybees

**DOI:** 10.3390/insects4040542

**Published:** 2013-10-18

**Authors:** Elizabeth Nicholls, Natalie Hempel de Ibarra

**Affiliations:** Centre for Research in Animal Behaviour, School of Psychology, University of Exeter, Perry Road, Exeter, EX4 4QG, UK; E-Mail: E.K.Nicholls@ex.ac.uk

**Keywords:** pollen, honeybee, learning, PER, conditioning, reinforcement

## Abstract

The function of pollen as a reward for foraging bees is little understood, though there is evidence to suggest that it can reinforce associations with visual and olfactory floral cues. Foraging bees do not feed on pollen, thus one could argue that it cannot serve as an appetitive reinforcer in the same way as sucrose. However, ingestion is not a critical parameter for sucrose reinforcement, since olfactory proboscis extension (PER) learning can be conditioned through antennal stimulation only. During pollen collection, the antennae and mouthparts come into contact with pollen, thus it is possible that pollen reinforces associative learning through similar gustatory pathways as sucrose. Here pollen was presented as the unconditioned stimulus (US), either in its natural state or in a 30% pollen-water solution, and was found to elicit proboscis extension following antennal stimulation. Control groups were exposed to either sucrose or a clean sponge as the US, or an unpaired presentation of the conditioned stimulus (CS) and pollen US. Despite steady levels of responding to the US, bees did not learn to associate a neutral odour with the delivery of a pollen reward, thus whilst pollen has a proboscis extension releasing function, it does not reinforce olfactory PER learning.

## 1. Introduction

The neural pathways underlying learning with sucrose rewards are well understood. Bees easily form associations between sucrose and visual, olfactory and tactile stimuli [1,2], leading to memories that last over extended time periods [3,4,5,6]. The sucrose reward resembles floral nectar rewards collected by bees whilst visiting flowers, and the ecological validity of such experimental work has been widely accepted [7,8,9]. Flowers also offer pollen as a food source for bee larvae and reproducing females, which is collected by non-reproducing workers in social bees and by female solitary bees. As with nectar, pollen is often assumed to be perceived by bees as a reward of varying profitability, based on the differences in protein and amino acid content between pollen from different plant species [10,11,12,13]. However, there is surprisingly little evidence to support such an assumption. Although bees form preferences for particular pollen types [14,15,16,17], it is still unknown how these preferences are formed and what bees might learn during pollen collection. Unlike sucrose solutions or nectar, pollen is not ingested by foraging bees, thus raising the question of how pollen could act as a reward for learning floral cues.

Bees have been shown capable of learning to associate pollen odour with a sucrose reward [18,19], and more recent evidence suggests that freely flying bees are able to associate pollen itself with both visual [20] and olfactory stimuli [21]. Pollen is a complex stimulus comprising of visual, tactile and multiple chemical components, but one would assume that gustatory perception of pollen is involved in pollen-mediated learning. Bees have gustatory receptors located on the antennae, mouthparts and tarsi [22] and so are likely to have ample opportunity to sample the taste of pollen during the process of collection. Indeed, foragers have been observed to touch the anthers of flowers with their antennae during pollen collection and even prior to landing [23].

Whilst free-flying experiments closely resemble the natural foraging situation, given the multiple sites of pollen contact during collection, such methods do not permit identification of the exact reinforcing components of the pollen reward. Scheiner *et al*. [24] have shown that stimulation of the antennae with hand-collected pollen grains in restrained honeybees leads to an extension of the proboscis, an appetitive reaction termed the proboscis extension response (PER). The PER can also be elicited when the antennae are touched with sucrose solution, and this has frequently been used as the unconditioned stimulus (US) in the PER conditioning paradigm. The unconditioned stimulus (US) is paired with the presentation of a neutral odour (conditioned stimulus, CS) and following a few training trials, presentation of the previously neutral odour alone becomes sufficient to elicit the PER over long periods of time [25,26]. Here we adapted this robust learning paradigm, which permits close control over the site and timing of stimulus and reward delivery, to test whether stimulation at the antennae with pollen, presented either as dry powder or in water solution, reinforces learning of a neutral odour. Whilst previous attempts have been made to condition the PER using pollen as a reward [21,27] and have reported some success, these studies lack the appropriate controls necessary to rule out the influence of non-associative effects resulting from repeated stimulation, which may also lead to an increase in responsiveness to the CS. To fully demonstrate that explicit pairing of the CS and pollen reward leads to an increase in responsiveness to the olfactory CS, as a result of bees having learnt the predictive relationship between the two, we included control groups which either received unpaired presentations of the CS and pollen US, or were subject to mechanical stimulation alone.

## 2. Materials and Methods

### 2.1. Subjects

Departing honeybee foragers (*Apis mellifera*) were collected from colonies located at Washington Singer Laboratories, University of Exeter. Individual bees were captured in small glass vials and placed on ice. Immediately after they stopped moving, bees were transferred into metal harnesses which permitted free movement of the antennae and proboscis [25]. Each subject was fed 30% (w/w) sucrose solution until satiated and then left undisturbed for 20 h until the start of experiments.

### 2.2. Stimuli

The conditioned stimulus (CS) was 1-hexanol (98% purity, Sigma Aldrich, Gilliam, Dorset, UK) diluted in mineral oil to 2.5 M. 20 mL of the odour solution was placed in a 60 mL glass bottle which was connected to an air pump via silicone tubing [28,29]. The air stream was gated by a valve activated by a programmable logical controller, to ensure delivery of uniform odour puffs. The odour stream was directed frontally at the head of the bee, and removed via a constant air stream emitted from an extractor system located behind the animal. The pollen reward consisted of commercial honeybee collected pollen (Werner Seip, Ebersgöns, Germany) ground to a fine powder, and either delivered as a very thin layer of dry powder on a small, disposable eyeshadow applicator sponge, or mixed with water (30% pollen w/w). Pollen solutions were passed through filter paper to remove the larger clumps which were found to stick to the antennae and interfere with the experiment. The sucrose reward was a 30% (w/w) sucrose and water mixture which was also filtered. All pollen stimuli were freshly prepared before each training session. We did not observe any clogging of the antennae (and mouthparts in the second experiment) after repeated stimulation with the pollen stimuli.

### 2.3. Pre-Training Sensitivity Test

Prior to conditioning, bees were tested for their motivational state and sensitivity to pollen. The antennae of each subject were touched first with water, with those individuals displaying proboscis extension being permitted to drink until satiated. Antennae were then touched with 30% pollen-water solution, followed by water to separate the application of stimuli, 15% sucrose, water again, and finally 30% sucrose, each delivered at five minute intervals. For bees in the dry pollen experiment, 30% pollen-water solution was replaced by dry pollen grains, delivered via a small sponge (1 cm length) which was replaced following each experimental block. The criteria for inclusion in a conditioning experiment was that bees must respond to both pollen (or pollen in water) and 15% sucrose (40% of bees on average). Conditioning began twenty minutes after the sensitivity test to allow any potential effects of sensitization to sucrose to subside [30].

### 2.4. General Conditioning Protocol

An individual bee was placed in the experimental arena at a distance of 4.5 cm from the odour delivery tube and left to habituate for 15 s prior to the start of the CS. The odour (CS) was delivered to the antennae for three seconds alone and overlapping for one second with the unconditioned pollen or sucrose stimulus (US). US delivery then continued for a further two seconds. Depending on experimental design, the US was either presented first to the antennae and then the proboscis (experiment with pollen-water solution) or to the antennae alone (dry pollen conditioning experiment). Following US presentation, bees were left in the arena for a further 15 s and then removed for an inter-trial interval of ten minutes until the next conditioning trial or test.

### 2.5. Dry Pollen Conditioning

Dry pollen emits odours that could potentially interfere with the CS presentation or stimulate rewarding pathways (spontaneous US responses or second-order conditioning effects). To achieve a uniform presentation of pollen odour in the treatment and control groups, one clean and one pollen-coated sponge were taped together in a cross formation. Bees received either stimulation with the pollen sponge (pollen group) or the clean sponge (control group). In addition, a small petri dish (5.5 cm diameter) containing *ca*. 3 g of pollen was also placed between the bee and the syringe delivering the CS to create a standardized olfactory environment in the arena. A third group of bees received 30% sucrose as a control, which was presented using a cocktail stick. Following six conditioning trials bees received an unrewarded test trial, in which only the CS was presented. Proboscis extensions were noted both during the presentation of the CS alone and during US delivery.

Typically in PER conditioning bees are rewarded with a toothpick dipped in sucrose solution. Stimulating bees with a dry toothpick alone does not reinforce learning [31], hence mechanical stimulation can be ruled out as a potential component of rewards delivered in this manner. Stimulation with softer substances, such as sponge, have yet to be tested. Also the presence of pollen odour in the arena could have influenced the behaviour and learning performance of bees, potentially leading to spontaneous or unconditioned learning responses. We conducted a control experiment, in which only half of the bees were exposed to pollen dish odour in the arena whilst being conditioned with either a clean sponge (mechanical stimulation only) or with sucrose (2 × 2 design). To remove the pollen odour from the arena for the other half of bees, the pollen dish was covered with a lid and any odour was removed via the extracting system.

Bees received six conditioning trials and two unrewarded test trials with an inter-trial interval of 10 min. In addition to recording proboscis extensions during CS and US presentation, we also recorded if the proboscis was extended prior to CS delivery, to test whether bees in the pollen-dish condition were more aroused than those not exposed to the pollen dish. In Test 1 bees experienced the same conditions as in training (*i.e*. dish was uncovered for bees conditioned with the arena pollen odour present and covered for bees conditioned without it). In Test 2 the pollen dish was covered for all bees, to test whether pollen odour might act as a contextual cue for the recall of the CS-US association. If so, we expected to see a drastic decline in response between Test 1 and 2 for bees exposed to pollen odour during conditioning, but not in the other bees. Tests were delivered in a fixed order.

### 2.6. Antennal Sensitivity to Pollen in Water Solution

To remove the effect of pollen odour completely and to test a different pollen delivery method we dissolved pollen in water. It was necessary to determine what constitutes an appropriate dilution of pollen and water to use in conditioning experiments. Though bees may not be able to detect the presence of pollen in weaker solutions, the strongest concentration may not necessarily be that which is most preferred by bees. Based on Page *et al*.’s [32] method, for assessing the gustatory responsiveness of honeybees to sucrose, we tested antennal sensitivity to solutions containing increasing concentrations of pollen. Samples ranged from 0.1% pollen (w/w) to 30% pollen (w/w) and were all passed through filter paper in order to remove the largest grains (9 micrometre maximal pore size). Higher concentrations were found to contain too much dense matter which would clog the bee’s antennae. The strongest solution we were able to produce which didn’t have this effect was a mixture of 30% pollen and water (w/w).

Bees returning to the hive were collected, with pollen and non-pollen foragers indentified by the presence/absence of corbiculae loads. Thus it was possible to examine whether differences exist between the two forager types in terms of antennal sensitivity to pollen, as has previously been suggested by Scheiner *et al*. [24] for bees stimulated with dry pollen. As in PER conditioning experiments, bees were restrained in metal harnesses and 15 bees of each forager type were tested in parallel. A small piece of tape was added to the base of tubes containing pollen foragers. This was hidden from the view of the experimenter, to avoid any potential bias in the coding of behaviour.

Bees were tested with ascending pollen concentrations from pure water to 30% pollen, with proboscis extension to antennal stimulation recorded on each trial. Solutions were delivered via a toothpick and water via a syringe. On the first trial, in which bees were stimulated with water alone, individuals were permitted to drink until satiated, in an attempt to minimise the influence of thirst on the response to the pollen-water solutions. To avoid cross-contamination, the antennae were stimulated with water prior to each pollen stimulation. The inter-trial interval was ten minutes. At the end of the experiment, the antennae were stimulated with 30% sucrose, to demonstrate that bees were still motivated to feed, and that any decline in response to the pollen-water solution was not the result of a general lack of motivation or fatigue.

### 2.7. Conditioning with a Pollen-Water Solution

To determine whether pollen in solution can reinforce learning, three groups of bees were tested. One group were exposed to a forward pairing of the olfactory CS and 30% pollen-water (w/w) US. A control group, which received unpaired presentations of the CS and pollen-water US, was included to rule out the possibility that any observed increase in response to the CS was simply due to non-associative effects of repeated antennal stimulation. As a positive control, a third group received forward pairings of the same olfactory CS with 30% sucrose as the US. This group served as a baseline against which to compare learning performance.

In forward paired trials, the odour (CS) was delivered first followed by the US (30% pollen-water or 30% sucrose solution), with an overlap of one second (as in the dry pollen conditioning experiments). The US was first presented to the antennae to elicit the proboscis extension response (PER) and then to the proboscis for consumption, similar to standard PER conditioning experiments. In unpaired trials, the pollen-water US was delivered first for three seconds (backward order of presentation). Following a delay of ten seconds, the odour (CS) was presented for four seconds. This was done for the first three trials of training. In the last three trials all bees received a forward pairing of the CS with sucrose reward (30% solution) to control for the changes in motivation or state of bees in the pollen-rewarded groups.

### 2.8. Statistical Analysis

Subject responses were scored as binary variables. To scrutinise the learning effects, bees that responded to the CS odour on the first trial were excluded from the analysis, since it could be assumed that such bees have a pre-existing appetitive memory for the CS. GEE (Generalized Estimating Equation) modeling was used to compare the acquisition curves of bees trained with different unconditioned stimuli [33]. The GEE approach is an extension of the Generalized Linear Model (GZLM) which permits a non-normal distribution of the dependent variable (*i.e*., binary distribution) and accounts for repeated measurements of the same individual [34]. Response to the CS was coded as the response variable with treatment and conditioning trial included as factors. Significance tests were based on Wald approximations of the likelihood ratio test. Post-hoc least significant difference contrasts (LSD) were used to compare treatment groups. Bees’ performance in the unrewarded tests was compared using Generalised Linear Models (GZLM).

## 3. Results and Discussion

### 3.1. Bees Did not Associate the CS with a Dry Pollen US

Whilst evidence exists to suggest that foraging bees are capable of associating both visual and olfactory stimuli with pollen reward, its reinforcing properties remain unknown. Only young honeybees in the first days after enclosure consume pollen; it is rarely found in the gut of foraging bees [35]. Thus, pollen is likely to activate non-ingestive reward pathways. A prime candidate for sensing pollen properties are the antennae which possess olfactory, gustatory and mechanical receptors and frequently come in touch with pollen during pollen collection. The olfactory PER conditioning paradigm was adapted as a method which allows precise control over reward delivery, as compared to methods involving freely flying, pollen-collecting bees. Three groups were conditioned with either a pollen-coated sponge (pollen group), clean sponge (control, mechanical stimulation only group) or 30% sucrose solution (sucrose group), predicting that, if pollen acts as a reinforcer, bees in the sucrose and pollen groups would show higher responses than the control group in the unrewarded test.

**Figure 1:**
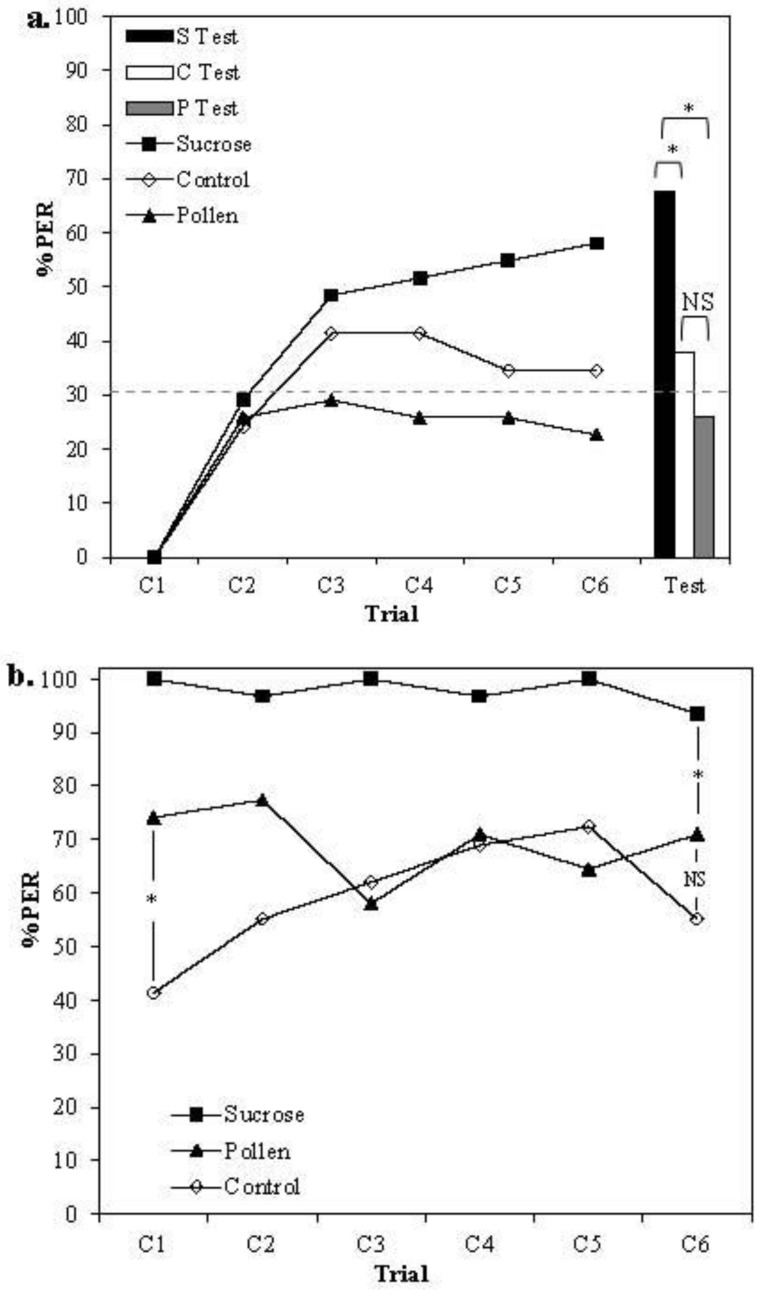
(**a**) Acquisition curves and (**b**) PER response to US stimulation of bees rewarded at the antennae only with 30% sucrose solution (Black squares, *n* = 31), dry pollen (Black triangles, *n* = 31) or a clean sponge (Open diamonds, *n* = 29). Bars represent the number of bees responding to the conditioned stimulus (CS) in the final, unrewarded test (Black = Sucrose, White = Control, Grey = Pollen). Dashed line represents the overall spontaneous response to the CS on the first trial for all bees tested. Asterisks denote significant differences between treatment groups (** *p* <0.001 * *p* <0.05).

In all groups, an increase in response to the CS was observed after the first conditioning trial (Figure 1a), but only the sucrose group reached acquisition levels well above the spontaneous response rate. This indicates that bees in the pollen group did not learn the task. The main effect of treatment was significant (GEE, Treatment *X^2^_2_* = 6.480, *p* = 0.039), and post-hoc analysis revealed a significant difference between the pollen- and sucrose-rewarded groups (LSD contrast *p* *=* 0.009). Likewise, in the unrewarded test, sucrose-rewarded bees responded significantly more than those in the other groups (GZLM, Treatment *X^2^_2_* = 10.919, *p* = 0.004; LSD contrast, Sucrose *vs*. Pollen *p* < 0.001, Sucrose *vs*. Control *p* = 0.015). There was no significant difference in responding between control bees receiving mechanical stimulation only and those rewarded with pollen (LSD contrast, Control *vs*. Pollen *p* = 0.311). Altogether, these results suggest that dry pollen did not reinforce learning.

To examine whether differences in US responsiveness between groups might explain the lack of learning in pollen-rewarded bees, responses to antennal stimulation with the US were compared (Figure 1b). The response of bees stimulated with 30% sucrose remained consistently high throughout the course of the experiment (>90%). Pollen-rewarded bees also showed consistent responses, albeit at a lower level (60%–70%). Whilst pollen initially elicited a higher proportion of proboscis extensions than mechanical stimulation alone (*ca.* 70% for pollen *vs*. 40% for clean sponge, GZLM, Trial 1, Treatment *X^2^_2_* = 6.349, *p* = 0.042, LSD contrast *p* = 0.016), over the course of the first three exposures, responses converged to the same level (GZLM, Trial 6, Treatment *X^2^_2_* = 6.349, *p* = 0.042, LSD contrast *p* = 0.20). Thus, a change of motivation in pollen-rewarded bees does not explain the lack of learning in this group.

The high level of CS and US responses to the stimulations with a clean sponge (control group, mechanical stimulation only) were unexpected. One potential explanation is that bees were aroused by the presence of pollen odour in the arena, which sustained their spontaneous responses throughout the experiment. We therefore conducted a control experiment in which the pollen dish odour was removed for half of the bees that were conditioned with either a single clean sponge or sucrose. We predicted that bees exposed to the pollen dish odour in the arena would show a higher rate of CS and US responses as compared to bees without pollen dish odour, if they were more aroused.

**Figure 2:**
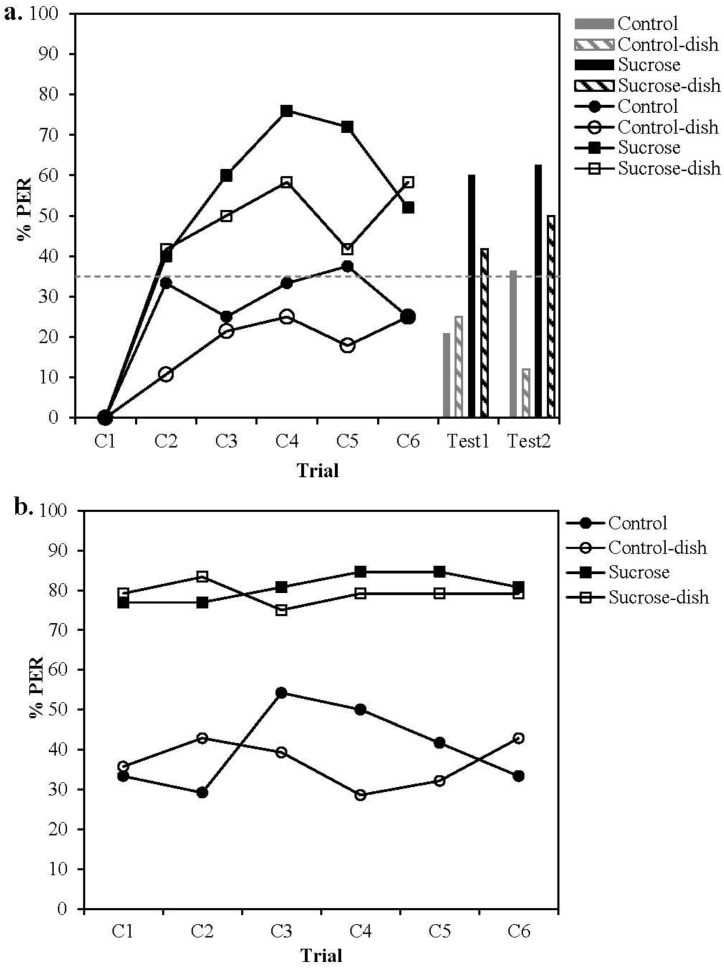
Proportion of bees responding to the CS (**a**) or US (**b**) on each trial. The US was either 30% sucrose solution (Squares) or a clean sponge (Circles). Bees were either exposed to an open dish of pollen during conditioning (Open shapes, *n* = 53) or a closed dish (Black shapes, *n* = 48). Bars represent the number of bees responding to the CS in the unrewarded tests. (Black bars=Sucrose, Grey bars=Control, Striped=Exposed to pollen; Striped= Non-exposed). In Test 1 olfactory conditions were matched to those experienced during training and in Test 2 the pollen dish was covered for all bees. The dashed line represents the overall spontaneous response to the CS on the first trial for all bees tested. Asterisks denote significant differences between treatment groups (** *p* < 0.001 * *p* < 0.05).

However, this was not the case, as can be seen in Figure 2. The statistical analysis confirmed that the presence of the pollen dish odour had no impact on the overall level of acquisition in any of the groups (GEE, Condition *X^2^_1_* = 2.680, *p* = 0.102; Condition x Treatment *X^2^_1_* = 0.074, *p* = 0.785). Whilst sucrose-rewarded bees learnt the association and outperformed the control bees receiving mechanical stimulation with a clean sponge (Figure 2a GEE, Treatment *X^2^_1_* = 17.659, *p* < 0.001), control bees showed little change in their response to the CS over time, remaining at the level of spontaneous responses, similar to the previous experiment. We also recorded whether bees extended their proboscis prior to the delivery of the CS, to test whether bees in the pollen-dish condition were more aroused than those not exposed to the pollen dish. The presence of the pollen dish had no effect on the proportion of bees exhibiting PER prior to CS delivery. For both control groups (presence *vs*. absence of pollen dish), on average *ca.* 1% bees exhibited PER prior to CS delivery. Likewise, on average 6% of sucrose bees exposed to the pollen dish exhibited PER prior to CS delivery compared to 10% of sucrose rewarded bees who did not experience the pollen dish, suggesting that sucrose-rewarded bees were slightly more aroused than those in the pollen group. Again this result indicates that pollen odour did not generate an increased level of sensitivity or arousal in bees.

Two unrewarded tests were conducted. The first test resembled the conditioning trials but excluded the US delivery. We predicted that bees exposed to pollen dish odour during training would respond more strongly to the CS in the first test, where pollen dish odour was present, than in the second test, where it was absent. Performance in bees not exposed to pollen dish odour during training should decrease between these two tests given the absence of the US.

Sucrose-rewarded bees in both conditions showed significantly higher responses than control bees (Figure 2a, GEE, Treatment *X^2^_1_* = 17.861, *p* < 0.001), but the overall difference in responding to the CS in bees that experienced the pollen odour *versus* those that did not is not significant (GEE, Condition *X^2^_1_* = 3.459, *p* = 0.063). This is likely to be due to different patterns of responses. The control bees that had experienced pollen dish odour during training decreased their responses between the first and second test, as predicted. Contrary to expectations, the sucrose-rewarded bees that experienced pollen dish odour increased their responses in the second test which indicates that pollen dish odour interacted to some degree with the CS presentation, presumably making it easier for bees to detect the CS in the second test. The sucrose-rewarded bees did not change their response between tests. The control bees that were not exposed to pollen dish odour increased their responses in the second test, contrary to predictions, which indicates that repeated mechanical stimulation with a clean sponge could sustain spontaneous response levels over the duration of the experiment. Finally, no significant interaction between olfactory conditions experienced during training, and responding in each test were observed (GEE, Test × Condition, *X^2^_1_* = 1.193, *p* = 0.275), suggesting that the absence of this cue does not affect recall in those bees trained in the presence of pollen odour.

The US responses were as expected, in that bees stimulated at the antennae with sucrose showed a higher level of responding than those stimulated with a clean sponge (Figure 2b, GEE Treatment *X^2^_1_* = 28.55, *p* < 0.001). The presence of the pollen dish odour had no significant effect on responding to the US (GEE, Condition *X^2^_1_* = 0.075, *p* = 0.784). Overall responding to antennal stimulation remained constant throughout the course of the experiment (Trial *X^2^_5_* = 2.583, *p* = 0.764; Treatment × Trial *X^2^_5_* = 3.795, *p* = 0.579).

In summary, we did not find evidence of increased arousal due to the presence of pollen dish odour in the arena. Rather it seems that simply stimulating the antennal mechanoreceptors of hungry bees may be sufficient to elicit proboscis extension in some cases. The findings of the control experiment support the conclusion reached for the first experiment, which is that responses seen in the pollen and control group do not reflect learning of the CS.

Since pollen stimulation did not lead to reinforcement of behaviour, perhaps gustatory receptors on the proboscis, maxillae or mandibles may be more important than those on the antennae for receiving reward input. Sandoz *et al*. [36] suggested that for sucrose conditioning, antennal input predicts reward delivery, but that proboscis input is more important and encodes more information about the nature of the reward. Mustard *et al*. [37] found that the proboscis is more sensitive to caffeine (in solution with sucrose) than the antennae, indicating that the proboscis may also be important for the detection of bitter tastes.

When applying dry pollen directly to the proboscis we observed that after several trials, grains would adhere to the surface of the mouthparts and inhibit proboscis extension, the very measure of learning used in the olfactory conditioning paradigm. When bees were restrained, they were unable to clean the proboscis with the forelegs as they are observed to do when foraging naturally [23,38]. Whilst both the proboscis and antennae could be responsible for sampling pollen during collection, the legs are involved in gathering pollen from the anthers of flowers, grooming excess grains from the head and body and packaging grains into the corbiculae. The tarsi also possess gustatory receptors, but the role they might play in the reinforcement of pollen-rewarded behaviour was not investigated here.

### 3.2. Presence of Pollen in Water Solution Was Detected by Bees, but Did not Reinforce CS Learning

**Figure 3:**
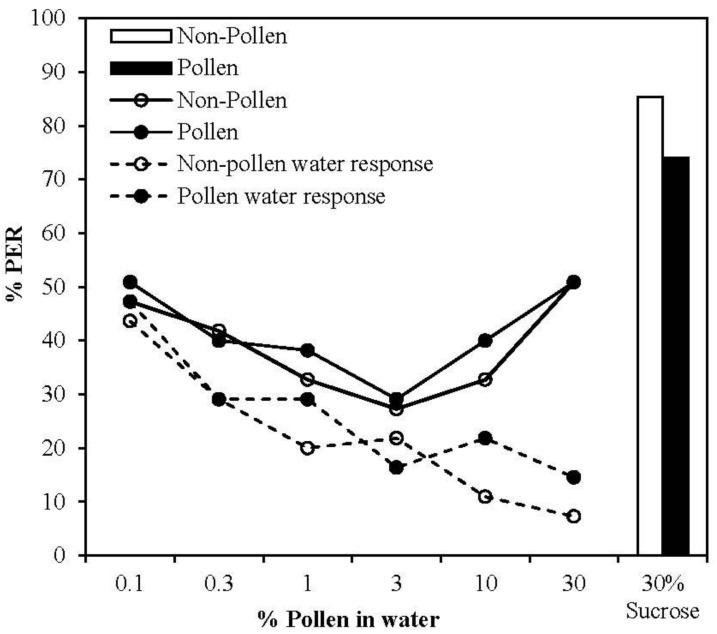
Proportion of bees extending the proboscis to antennal stimulation with a series of pollen-water mixtures, tested in order of ascending pollen concentration. Bees were separated according to forager type (Black circles = Pollen foragers, *n* = 53 Open circles = Non-pollen foragers, *n* = 55). Bars represent the response to a final stimulation with 30% sucrose (White = pollen foragers, Black = non-pollen foragers). The dashed lines represent bees’ responses to water stimulation prior to the stimulation with each pollen concentration (Black triangles = pollen foragers, Open triangles = non-pollen foragers).

Using a gustatory assay, we established whether bees could sense pollen dissolved in water. An initial decline in response to stimulation with both pure water and pollen-water mixtures ranging from 0.1% to 3% pollen (w/w) was observed (Figure 3). When stimulated with 10% and 30% pollen respectively, the level of responding to the pollen-sucrose mixture increased and was significantly different from the response to water alone (GEE, 10% Pollen, Treatment *X^2^_1_* = 38.460, *p* < 0.001; 30% Pollen, Treatment *X^2^_1_* = 28.30, *p* < 0.001). Thus 30% pollen was selected as the concentration to be used in subsequent conditioning experiments. This U-shaped pattern of responding likely stems from the fact that initial levels of responding to weak pollen solutions (0.1%–3% pollen) was in fact driven by the water component of the reward, with pollen compounds remaining undetected at such concentrations. A similar decline in responding was observed in both types of trial as individuals habituated to this stimulus. At concentrations of 10% pollen or greater, bees were clearly able to detect pollen compounds in solution, as evidenced by the significantly higher level of responding compared to trials with water alone. Pollen and non-pollen foragers responded in a similar manner to antennal stimulation with the various solutions over the course of the experiment, suggesting there is little difference between forager types in terms of sensitivity to pollen in water solution.

**Figure 4:**
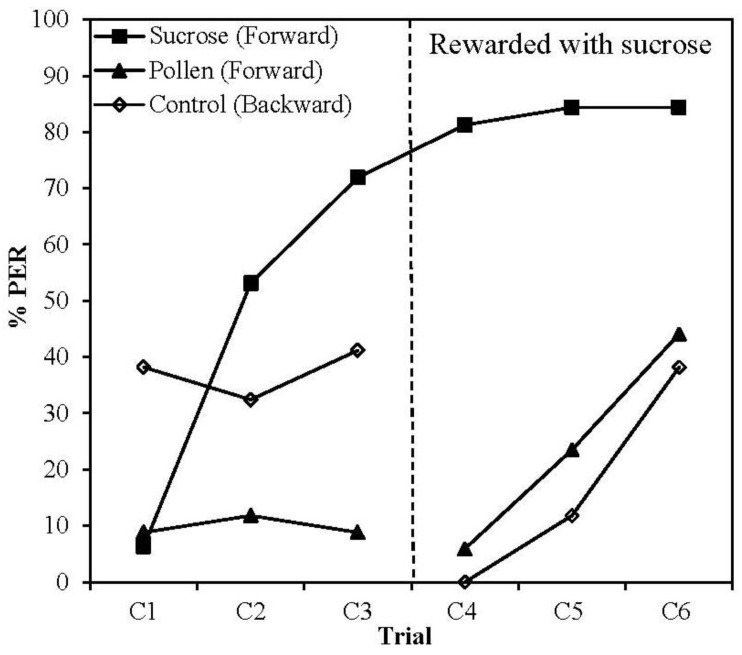
Proportion of bees extending the proboscis to a previously neutral odour following pairing with different unconditioned stimuli. For trials 1–3, the US was either 30% pollen (w/w) solution (Black triangles, forward paired, *n* = 34, White diamonds, backward paired, *n* = 34) or 30% sucrose (w/w) solution (Black squares, *n* = 32). All bees received 30% sucrose on trials 4–6. The US was applied to both the antennae and proboscis.

When we conditioned bees with the pollen-water solution, applied to both antennae and proboscis, we did not find evidence for CS learning (Figure 4). Sucrose-rewarded bees learnt the CS, as expected (GEE, Treatment *X^2^_2_* = 14.393, *p* = 0.001; Treatment × Trial *X^2^_4_* = 8.894, *p* = 0.001), showing a significant increase in response to the CS between trials one and three (Sucrose, T_1_ *vs*. T_3_, *p* < 0.001). Bees rewarded with a pollen-water US did not show any change in response over these three trials (Pollen (paired), T_1_ *vs*. T_3_, *p* = 1.000; Control (unpaired), T_1_ *vs*. T_3_, *p* = 0.479), suggesting that pollen failed to support learning of the predictive relationship between CS and US delivery. Note that bees exposed to unpaired CS-US presentations (control) experienced the US first, which led to consistent levels of arousal responses, *i.e*., bees kept extending their proboscis during the CS presentation, leading to a higher (but unchanged) response level during three trials of training.

From the fourth trial (C_4_) onwards, bees in all groups were rewarded with sucrose and received forward pairings of the CS and US. On the fourth exposure to the CS (prior to administering sucrose reward in C_4_) bees previously conditioned with pollen-water US (forward pairing) still did not respond to the CS, confirming that bees in this group had not acquired an association between the CS and pollen US. Bees that were previously rewarded with pollen (control and pollen groups) showed a significant increase in responding to the CS over the course of the final trials (GEE, Trial *X^2^_1_* = 19.384, *p* < 0.001; Pollen *p* = 0.001; Control *p* = 0.005), thus demonstrating their capacity to learn the contingency between the CS and sucrose US.

The current findings contrast with those of earlier studies in which pollen rewarded learning was also investigated using the PER paradigm. Grüter *et al*. [27] and Arenas and Farina [21] claim that following few pairings of an olfactory CS and pollen US delivered to the antennae and proboscis [27] or proboscis only [21] bees would learn to respond with PER to the CS alone. Whilst it is possible that methodological differences may explain the discrepancy in results between these studies and our own [39,40], it is nonetheless clear that both previous studies lack the appropriate controls necessary to evince the formation of a CS-US association, and rule out that the possibility that the behavioural changes they observed were induced by non-associative effects resulting from repeated stimulation. To fully demonstrate that the explicit pairing of the CS and pollen reward causes an increase in response to the olfactory CS as a result of bees having learnt the predictive relationship between the two stimuli, it is crucial to include a control group where bees which receive unpaired presentations of the CS and pollen US and fail to show the same increase in responsiveness to the CS over time.

Arenas and Farina [21] did not perform any unrewarded tests in the PER experiments and group sizes differed strongly within each experiment which can affect between-group comparisons. The pollen used was hand-collected, but the application method was not described. Fresh floral pollen tends to be sticky and it could well be that it clogged the mouthparts, preventing the experimenters from reliably stimulating the proboscis from controlling US exposure time. In the study by Grüter *et al*. [27] it is unclear exactly how the pollen-water US, constituting 50%–70% pollen and 50%–30% water (w/w), was presented to antennae and proboscis, since we found that high concentrations of pollen in water (*i.e*., more than 30% (w/w)) results in a sticky mass that easily clogs the antennae. It is therefore unclear how, in Grüter *et al*.’s [27] study, the pollen-water mixture could have been repeatedly delivered to the antennae and proboscis without affecting bees. The reported increase in responding to the CS within three trials may have resulted from non-associative processes such as sensitization or simply due to repeated mechanical stimulation, as we found in this study. Controls required to demonstrate associative learning were absent in Grüter *et al*.’s [27] study, for example unpaired CS-US presentations or at least water-rewarded bees trained in parallel to the pollen-rewarded group.

## 4. Conclusions

Whilst pollen has been shown to reinforce learning in free flying bees, isolating the sensory and learning mechanisms involved in pollen-rewarded learning has proven more difficult. In the experiments presented here, we took advantage of a paradigm in which bees are restrained, permitting tighter control over stimulus delivery. The aim was to identify those sensory organs most likely to be involved in transmitting sensory information about the pollen reward, separate from other processes that may occur during pollen collection in free flying bees.

Antennal stimulation with dry pollen did not support learning of a contingent relationship between this US and an olfactory CS. Nor did bees learn to associate the CS with pollen when it was added to water and delivered in a soluble form. Our findings do not confirm claims [21,27] that pollen reinforces PER learning. Taken together with the lack of controls in those previous studies, our results render these claims unsubstantiated.

We observed steady levels of US responses to the pollen reward when the antennae were touched with pollen, which is in agreement with results reported by Scheiner *et al*. [24]. However, multiple pairings of this stimulation with a neutral odour did not lead to an increased response to the odour alone. Hammer and Menzel [6] suggest that stimuli which serve as the US reward are characterized by a releasing, modulating and reinforcing function, with the releasing and reinforcing functions of the sucrose reward having been shown to be dissociable [41]. Therefore it seems that under restrained conditions, pollen stimuli at the antennae may possess only a releasing function. 

Pollen collection involves a complex set of behaviours, and so restricting bees movement, as is necessary in the PER paradigm, may have precluded the performance of certain actions essential to the formation of an association between the CS and pollen. Here and in previous studies, pollen has been considered a form of appetitive reward and thus gustatory organs have been assumed to be implicated in the reward pathway. However, since pollen is not ingested, it may be that other receptors are involved, such as those sensitive to mechanical stimulation. In the honeybee, innervated setae (hairs) found on the corbiculae have been shown to be sensitive to the degree of mechanical displacement and have therefore been postulated to be involved in detecting changes in the size of the growing pollen load [42]. Bumblebees have been shown to adjust their handling time and grooming behaviour according to the availability of pollen at flowers [43,44,45] suggesting they are sensitive to feedback in terms of their pollen foraging success at each inflorescence.

The lack of olfactory learning under restrained conditions indicates that the behavioural context and methods used to examine learning should be carefully considered in future explorations of pollen-rewarded learning. Similar discrepancies between free-flying and restrained bees in terms of the ease with which bees can be conditioned have been observed for conditioning colour stimuli using the PER paradigm [46,47]. The question as to which factors and processes are necessary and sufficient to mediate pollen-rewarded learning in bees remains to be answered.
